# Systemic mistakes in hand hygiene practice in Ukraine: detection, consequences and ways of elimination

**DOI:** 10.3205/dgkh000244

**Published:** 2015-01-14

**Authors:** Iryna Klymenko, Günter Kampf

**Affiliations:** 1 Organization and Economy Department, P.L. Shupyk National Medical Academy of Postgraduate Education, Kiev, Ukraine; 2 Bode Science Center, Bode Chemie GmbH, Hamburg, Germany; 3 Institute of Hygiene and Environmental Medicine, University Medicine, Greifswald, Germany

**Keywords:** hand hygiene, hand disinfection, hand washing, hand care, compliance, healthcare settings, medical staff, Ukraine

## Abstract

**Aim:** Every year, millions of people around the world suffer from different infectious diseases, considerable part of which are hospital-acquired infections. WHO considers hand hygiene as a priority measure aimed to reduce the level of infection. We evaluated various aspects related to the situational behavior and prioritization regarding hand hygiene measures among the healthcare workers of Ukraine.

**Method:** Identification of system mistakes in hand hygiene was carried out first of all by direct and indirect observation of the activities of medical and pharmaceutical personnel in their everyday practice as well as during their participation in trainings on routine hand hygiene. Questionnaires also were used to estimate the level of hand hygiene compliance of participants of the study. During this period 112 training courses, 315 master-classes and presentations on proper hand hygiene were realized. The target audience included health care workers of medical centers, clinics, maternity hospitals, health care organizations and staff of pharmacies and pharmaceutical manufacturing enterprises in all regions of Ukraine. 638 respondents took part in anonymous survey on hand hygiene practice.

**Results:** The most common mistakes were to regard hand washing and hand disinfection equally, to wash hands before doing a hand disinfection, to neglect the five moments for hand hygiene and to ignore hand hygiene before and after wearing protective gloves. Practitioners, medical attendants, pharmacy and pharmaceutical industry workers highlighted the need for practical and understandable instructions of various hand hygiene procedures, including the clarification of the possible technical mistakes. This became a ground for us to create individual master classes on hand hygiene for each cluster of healthcare workers.

**Conclusions:** Changing hand hygiene behavior and attitude is possible by beginning to observe clinical practice and by involving healthcare workers in teaching and training.

## Zusammenfassung

**Ziel:** Jedes Jahr erleiden Millionen Menschen weltweit Infektionen, von denen ein beträchtlicher Anteil nosokomial ist. Die WHO betrachtet Händehygiene als wichtigste Maßnahme zu ihrer Prävention. Ziel dieser Studie war es, verschiedene Aspekte der Händehygiene zum situativen Verhalten und zur Präferenz von Maßnahmen bei Mitarbeitern im Gesundheitswesen der Ukraine zu erfahren.

**Methoden:** Die Bewertung grundsätzlicher Fehler in der Händehygiene wurde in erster Linie mittels direkter und indirekter Beobachtung vorgenommen. Hierzu wurden Tätigkeiten von medizinischem und pharmazeutischem Personal im Berufsalltag sowie während Schulungen zur Händehygiene beobachtet. Zusätzlich wurden Fragenbögen zur Bewertung der Händehygiene genutzt. Insgesamt wurden 112 Schulungen, 315 Fortbildungen und Präsentationen zur Händehygiene in die Auswertung eingeschlossen. Die Zuhörer waren Mitarbeiter aus Praxen, Kliniken, Geburtskliniken, Apotheken und Arzneimittelherstellern aus allen Regionen der Ukraine. An der anonymen Befragung zur Praxis der Händehygiene nahmen 638 Personen teil.

**Ergebnisse:** Die häufigsten Fehler waren die Betrachtung der Händedesinfektion und des Händewaschens als gleichwertig, das Waschen der Hände vor einer Händedesinfektion, das Vernachlässigen der 5 Momente der Händehygiene sowie das Ignorieren der Händehygiene vor und nach dem Tragen von Schutzhandschuhen. Alle Teilnehmer äußerten den Bedarf an praxisnahen und verständlichen Anweisungen zu den verschiedenen Händehygiene-Tätigkeiten einschließlich der Erklärung möglicher technischer Fehler in der Durchführung. Auf dieser Basis wurden individuelle berufsgruppenspezifische Masterklassen für Händehygiene erstellt.

**Fazit:** Das Verhalten und die Einstellung zur Händehygiene kann verändert werden, in dem zu Beginn die praktische Durchführung beobachtet wird und in dem die Mitarbeiter in die Schulung und Ausbildung eingebunden werden.

## Introduction

Hand disinfection though the simplest is the most effective and cheap measure to prevent the spread of many infectious diseases, particularly diseases caused by nosocomial pathogens [1]. It is impossible to solve one of the most important tasks of healthcare – patient safety – without understanding the importance of proper hand hygiene compliance, without promoting its implementation in the healthcare system at all financial, administrative and social levels [2].

As early as 2007 the Ministry of Health (MOH) of Ukraine has appointed measures to improve hand hygiene to be one of the most important means in preventing the spread of infections [3], [4]. In 2009, a team of specialists of MOH and the National Academy of Medical Sciences of Ukraine have developed the Methodic Recommendations “Surgical and Hygienic Hand Treatment of Medical Staff” and on September 21, 2010 this document was put into effect by the order of the Ministry of Health No. 798 [5]. At the same time Ukraine became a member of the WHO campaign on hand hygiene. It should be noted that although the level of compliance with the proper hand hygiene in many healthcare settings in Ukraine remains very low [2], [6], when the Order No. 798 was issued, the medical staff started to put more emphasis on hand hygiene.

As for hand hygiene, the term “compliance” means the adherence to hand hygiene guidelines. The generally accepted metric for monitoring and recording adherence (compliance rate) is the number of hand hygiene episodes performed by personnel divided by the number of hand hygiene opportunities [7]. Correspondingly, it seems appropriate to use the definition “noncompliance” when speaking about voluntary and unconscious avoidance or resistance of the pharmaceutical and healthcare professionals to hand hygiene measures, their mistakes and wrong priorities when they choose the methods of hand disinfection.

It is proved that the healthcare noncompliance of the workers with hand hygiene is the most serious obstacle in implementation and improvement of hand hygiene practice [8]. The staff noncompliance consists of three key elements: the neglect or failure to comply with proper hand hygiene rules, wrong priorities when choosing the methods and products for hand treatment and mistakes in carrying out the hand hygiene procedures [9]. According to the international reports, even in countries where the status of hand hygiene is very high and sufficient funding is allocated for its implementation, healthcare workers still routinely make mistakes in their hand hygiene practice [2], [9].

Undoubtedly it is mandatory to comply with hand hygiene guidelines [5], but as practice shows, it is not always enough to maintain hand hygiene at the appropriate level. It was found out through the surveys, questioning and trainings in the advanced training courses that almost 90% of the Ukrainian medical professionals and pharmacists make mistakes during the regular and timely hygienic procedures of hand washing, hand disinfection and hand care [6]. Even recognizing the need and importance of hand hygiene and having everything necessary for its proper implementation, the medical workers often do not notice when they make mistakes and what the essence of their mistakes is.

## Objective and methods

The low level of compliance with hand hygiene among health care and pharmaceutical workers became a valid reason for detection and systematization of the major mistakes that cause violation of the rules and guidelines of the proper hand hygiene (voluntary and unconscious noncompliance).

Our study lasted in the period 2009–2014. Identification of system mistakes in hand hygiene was carried out by:

- Direct and indirect observation of the activities of medical and pharmaceutical personnel in their everyday practice as well as during their participation in trainings on routine hand hygiene. About 90% of all technical mistakes in hands treatment were identified during observation.
- Questionnaires that were used to estimate the level of hand hygiene compliance of participants of the study. Questionnaires have become the main source of data for determining most of the situational mistakes.

During this period 112 training courses, 315 master-classes and presentations on proper hand hygiene were realized. The target audience included health care workers of medical centers, clinics, maternity hospitals, health care organizations and staff of pharmacies and pharmaceutical manufacturing enterprises in all regions of Ukraine. 638 respondents took part in anonymous survey on hand hygiene practice. 

Detection and analysis of these systemic mistakes allowed to recognize weak points in hand hygiene practice in Ukraine and to modify correspondingly training programs on proper hand hygiene: to improve present methods of training and to work out new ones.

Using the scientific literature and on the basis of data obtained from our study, we have divided the mistakes existing in hand hygiene practice into two groups (Table 1 (Tab. 1)):

1. Mistakes related to the situational behavior and setting of priorities.
2. Mistakes made directly during the hand treatment procedures (technical mistakes).

In the present work only the first group of the mistakes is analyzed. 

## Results and discussion

### Mistake 1: Equalization of hand washing with hand disinfection 

According to the study findings, it is one of the most common mistakes of this group [6]. For example, in a hand hygiene survey about 80% of the respondents used the term “wash” in relation to any method of hand disinfection, including the use of alcohol-based disinfectants for hand rubbing. The results of our survey showed that 13.8% of respondents are convinced that hand washing is as effective against pathogens as hand disinfection.

Hand washing is a mechanical hand cleaning using the simple soap and water. During this procedure visual contamination and sweat is removed from hands as well as the spore-forming bacteria. The transient microorganisms are only partly washed away from the hands [5]. 

After hand washing, the number of bacteria on hands is reduced by 90–99% (e.g. from 10 million to 100 thousand), which does not meet the requirements for the hand disinfection. Moreover, even the use of hand cleaners developed specifically to destroy the transient microorganisms (antibacterial soaps, washing lotions, etc.) usually does not provide the necessary level of reduction of pathogens. The only reliable method of hand decontamination is their rubbing with alcohol-based disinfectants. The number of colony-forming units (CFU) is reduced e.g. from 1 million to 10 when the alcohol-based disinfectants are used. Moreover, hand disinfection has some additional advantages over any other method of the hand washing: simple or antibacterial (Table 2 (Tab. 2)).

Use of alcohol-based hand disinfectants is generally considered not harmful to the skin, easy to use and most reliable because of wide microbiological spectrum with no resistance development [10], [11], [12]. Thus, hand washing does not have the same efficacy as hand disinfection. It is only one part of the three-component system of hand hygiene, which besides the hand washing also includes hand disinfection and hand care [10].

Hand washing can be the first choice only in well-defined cases, such as:

- Visibly soiled hands
- Hands contaminated with eggs of helminths and cryptosporidium oocysts

In case of contamination with spore-forming bacteria (e.g. *C. difficile*) hand washing should be performed after the hand disinfection.

### Mistake 2: Neglecting the main preconditions for hand hygiene

 This second common mistake includes all violations which staff makes when preparing for further treatment of their hands [5], [6], [10], [13], [14]. 

General requirements to the preconditions for hand hygiene include:

1. Clean and short cut nails (not longer than fingertips), the nails should not be broken or gnawed round. Most microorganisms located on the hands are concentrated at the fingertips. So long nails prevent thorough hand disinfection.
2. Absence of nail lacquer or false nails. Micro-cracks located on the lacquered nails and under the false nails create ideal conditions for colonization of the pathogens.
3. Refusal to polish the nails, preferably not only during the work shift, but also at home as dust on the hands after nail polishing is kept up to 4 days.
4. Absence of rings, bracelets, watches on hands (especially under the medical gloves) during hand treatment before surgical interventions, medical procedures and manufacture of medicinal products. The studies have shown that a large number of microorganism colonies are left on the jewelry and accessories, and on the skin under them after hand disinfection. Moreover, the items on the hands can cause injuries and skin irritation [10].

Nevertheless, according to the observation, the majority (69.7%) of health care workers neglected the main preconditions for hand hygiene. Mostly, we observed the presence of wedding rings on the hands of physicians and long varnished nails of female medical staff.

### Mistake 3: Washing of the visually clean hands/invariable hand washing directly before their disinfection

It is shown that not only the routine use of soaps, but also the frequent contact with water has a negative impact on the hand skin [15], [16], [17]. This is why the regular washing of the visually clean hands, especially just before their disinfection, is an unreasonable excessive load for the skin, which leads to its subsequent drying and irritation. Moreover hand washing with further drying requires additional time. 

Watching the routine work of staff in hospitals, we noted that 78% of the health care workers regularly wash visually clean hands before or instead of hand disinfection.

Hand washing procedure should be used only to remove visible dirt, and even in such cases hands should be thoroughly dried before hand disinfection [18]. It is recommended to perform hand disinfection not earlier than 10 minutes after contact with water [19].

### Mistake 4: Use of non-professional products for hand washing

When speaking about the hand washing procedure, special attention should be paid to the choice of soaps or other hand washing products. According to the data we obtained in the survey, 62.5% of respondents do not use professional hand washing products in their routine practice. Health care workers often choose low-quality soaps because of the influence of domestic hand washing market, which offers a huge number of non-professional cosmetic products and accompanies them with incompetent information from the mass media about the effectiveness and safety of these products [8]. 

Today liquid hand washing products that have weakly acid pH value of 5.5–6.0 and are based on the synthetic detergents are generally accepted as less harmful for the skin [13]. However, workers whose activity is related to the high hygiene requirements can use only professional washing products from this group. 

Professional hand washers should meet the following requirements:

- Absence of colorants and flavoring agents that can cause allergic reactions (colorants and flavors that are the components of the hand washers should confirm their status of non-allergenic in the relevant dermatological studies)
- Suitability for any skin type, including dry and/or sensitive skin [20]
- No influence on the effectiveness of hand disinfection (compatibility with hand disinfectants confirmed by the manufacturer) [21]

Upon the first request the manufacturer or distributor should provide the consumer with expert report or manufacturer declarations certifying all above mentioned characteristics of the washing product [22]. The cake soap being the potential reservoir for bacteria should not be used in the professional sphere [13]. Natural soaps (all components of such soaps are 100% made from the natural plants) can be used in the routine practice if they have confirmed all above mentioned professional characteristics.

### Mistake 5: Neglecting main causes (the five key moments) for hand disinfection

In our opinion it is the most critical situational mistake. Justifying their neglect of hand disinfection, healthcare workers point to different reasons: lack of time, too many causes for hand disinfection, unbelief in the efficacy of disinfection hand rubs etc. However, according to the results of our and foreign studies, even those workers who fully realize the importance of hand disinfection often do not know in what cases hand disinfection should be carried out unconditionally [23]. According to the results of our questionnaires and observations, 76% of health care workers miss at least one key moment for hand disinfection. Main causes (*the five key moments*) for hand disinfection are already specified by World Health Organization and by many specialists in Hygiene sphere [7], [16], [5], [23]. The five key moments are:

- Before patient contact
- Before an aseptic task
- After body fluid exposure risk
- After patient contact
- After contact with patient surroundings

In our investigation not only doctors and nurses took part but other workers of health care sphere also: pharmacists, cleaning personnel in hospitals, laboratory assistants. They pointed that their professional activity does not include all key moments mentioned above and it is difficult for them to determine their individual main grounds for hand disinfection. 

During the training course within the framework of our investigation we proposed the participants to work out their *individual (personal) plans* for hand hygiene. In these plans medical and pharmaceutical workers described their usual working day step by step including every hand hygiene procedure. Then the teacher checked these plans and discussed with the participants mistakes in choice and application of hand hygiene procedures. All needed corrections in plans were made during these discussions as well as on regular lessons after testing the plans in practice. Feedback that we received from the participants of the training course proved the efficacy of such approach. When a healthcare worker writes the personal plan on hand hygiene by himself, it makes him think more deeply and analyze every cause for hand treatment in his everyday activity. Such hygiene plan does not stay a theoretical base but becomes a real professional tool for a healthcare worker. 

### Mistake 6: Refusal of hand disinfection because of wrong ascription of dryness and irritation of hands to the effect of alcohol components contained in the hand disinfectants

According to the published information, many health care workers and pharmacists believe that the alcohol-based hand disinfectants are the most powerful skin irritants that cause allergic reactions and lead to dermatitis [13]. This opinion was confirmed by the results of the study, which we carried out among healthcare and pharmacy workers. 44.3% of respondents mistakenly identified the alcohol components in hand disinfectants to be harmful for hand skin. 53.5% of those who prefer hand washing specified that their skeptical rejection of the alcohol-based disinfectants was caused by the burning sensation during rubbing [8].

At the same time, existing studies of skin physiology, as already noted, have shown that classical hand washing is much more harmful than alcohol-based hand rubbing [12], [20], [24], [25]. Testing of the influence of ethyl, propyl and isopropyl alcohols on a hand skin has clearly shown that these chemical agents do not cause any allergic reactions and sensitization [12], [17], [24], [25]. In addition, the balanced composition of modern high-quality alcohol-based disinfectants includes the emollients – substances, which make skin soft and smooth and maintain skin protective function [26]. As for the burning sensation when using the alcohol-based disinfectants, it is only a sensory reaction that does not change the skin physiology. On the contrary, discomfort from the burning is the warning signal or the indicator of the already existing problems with the skin barrier mostly caused by frequent hand washing.

Insufficient understanding of the mechanism of action of alcohol-based hand rubs on a hand skin leads to the obvious negative consequences – almost harmless procedure of hand disinfection is rejected by the medical staff, instead healthcare workers frequently wash their hands with soap impairing the skin barrier. The detergents wash away the natural skin lipids, increase transepidermal water loss, which in turn causes dryness, roughness, exfoliation and eventually leads to dermatitis [19]. Resuming all mentioned above we can say that alcohol-based hand disinfectants are the products of choice because of their efficacy, skin perception and comfort. Moreover, according to the research results, regular hand rubbing by alcohol-based disinfectants instead of hand washing improves the skin condition [12].

### Mistake 7: Ignoring hand disinfection before and after wearing medical gloves

Speaking about proper hand hygiene practice, it is important to consider the situations related to the use of protective/medical gloves. Use of gloves gives the healthcare workers a false sense of confidence in the patients’ protection from the infections as well as in their own safety [27]. More then 60% of participants in our observations did not perform a disinfection of hands before putting on medical gloves, about 80% disregarded hand disinfection when taking the gloves off. 

Sure, the medical gloves are the essential and rational measure to stop the infection spread and protect the medical staff from contamination by the potential pathogenic agents. However, it is not enough to use just gloves to prevent transmission [23].

Possible perforation of the gloves during hygienic or aseptic manipulations makes the necessity of hand disinfection to be undoubted both before and after putting gloves on [5], [7], [10]. It is also proved that hands colonization, patient infection and contamination of the manufactured medicines can occur through the invisible microscopic holes in gloves that are put on hands [15]. According to the recent data, 3 out of 80 or 4 out of 120 new sterile gloves that were never used and were tested in accordance with EC 455-1 may be defective [19]. The perforation rate is 4% even after working in two pairs of gloves. In addition, when taking the gloves off, secondary hand contamination with the pathogens located on the gloves can occur. According to the research data, approximately 30% of healthcare workers have pathogens left on their hands after providing patient care [15]. Treatment of the disposable gloves on the hands with alcohol-based disinfectant is allowed only in situations that require frequent replacement of gloves, like blood sampling. In these cases, the gloves should not be punctured or be contaminated with blood or other fluids [5].

### Mistake 8: Interlaced use of two or more disinfectants with different exposure time

According to the recommendations of the Ministry of Health of Ukraine, full and constant supply of the healthcare staff with high quality hand disinfectants also includes the possibility to choose the hand rub products which are the most suitable for them [5]. However, this choice does not mean the interlaced use of two or more hand rubs for surgical hand disinfection if these products have different exposure time. According to the research data, if the surgery unit or some operation area has hand disinfectants with different time of antimicrobial action (1.5 min, 3 min, or 5 min), it increases the risk of confusion and use of the product with greater exposition within a very short time [28]. Today we find in at least every second operating room in Ukrainian hospitals two or more hand disinfectants with different application times. Of course Ministry of Health of Ukraine pays special attention to close compliance with the instructions/guidelines on the use of any hand disinfectants [5], but for alternation use it seems to be expedient to choose hand rubs that have the same exposition for surgical disinfection.

### Mistakes 9 and 10: Working or giving a permission to work to healthcare workers with the damaged hands skin. Neglecting the hand care

Based on the mentioned in the 6^th^ mistake paragraph it is evident that the part of workers who complain of the burning sensation when using alcohol-based disinfectants, are the workers who have pre-damaged or damaged hand skin. Our survey shows that 66.7% of respondents had one or more types of problems with the skin on their hands: dryness (43.7%), roughness (11.5%), redness (7%), peeling (4.6%) and itching (5.2%). Moreover, 16.8% of participants indicated that they perform their routine clinical work in spite of sometimes serious damage of hand skin such as erosion (3%), dermatitis (10.3%) or atopic eczema (3.5%). Identified high rates of hand skin problems among the healthcare workers connected with another mistake: work or giving a permission to work to the staff with damaged hand skin or even skin disease [8].

Poor state of a skin makes the disinfection procedure senseless, because hands with damaged skin create the ideal conditions for reproduction of pathogens. In other words, it is impossible to disinfect efficiently sore or irritated hands [10], [15], [16]. Healthcare workers and managers should remember that the work or permission to work to the staff with damaged hand skin can be the reason of infection both for the employees and patients.

As a result, maintaining the skin in a healthy condition is not a luxury but a personal professional responsibility of the healthcare professionals. In order to successfully fulfill this responsibility medical professionals should consistently, regularly and correctly use hand care products, which are dermatologically tested and approved for use in the medical and pharmaceutical fields [20], [21], [22].

However, according to our research, in practice less then 50% of medical and pharmaceutical staff regularly takes care of their hand skin.

### Mistake 11: Incorrect processing and filling of dosing containers

In daily hand hygiene practice in the healthcare settings it is very important to use suitable dosing devices to release the hygiene products as well as suitable pocket bottles with disinfectant [5]. Dispensers and bottles must fulfill special requirements that prevent the colonization of microorganisms [10], [29]. The ministry of Health of Ukraine specifies the following requirements for the maintenance of dosing devices and bottles [5]:

- Do not refill incompletely empty dosing devices with hygiene products
- Dosing devices for hand hygiene products have to be carefully cleaned and disinfected before each refill
- Completely empty bottles for hand treatment products should be refilled only in aseptic conditions
- It is recommended to use disposable containers.

Our observation showed that the above mentioned recommendations are often not taken into consideration by the medical staff in hospitals and especially polyclinics (up to 90%). 

An overview of situational mistakes that we have determined during our study is presented in Table 3 (Tab. 3). It confirms that medical and pharmaceutical workers still have a rather low level of knowledge about proper hand hygiene. 

## Conclusions

During the study, except for observations, surveys and questioning, the regular theoretical and practical lessons on proper hand hygiene were conducted for the healthcare workers. These lessons included explanations of how to prevent most of possible mistakes during the hand disinfection, training on proper hand rubbing and working up of individual plans for hand hygiene. Results of the study once again supported the statement of famous professionals in healthcare area that the improvement of the medical and pharmaceutical staff awareness concerning the proper hand hygiene is the key to their compliance with the corresponding measures.

Most of the medical and pharmaceutical workers who were involved in the study expressed a desire to receive more information on hand hygiene. However, the expected information differed depending on the type of their activity. The experts-managers expressed a desire to receive more scientifically based information on the proper conduct, microbiological efficacy, economic advantages and safety of hand hygiene measures. Practitioners, medical attendants, pharmacy and pharmaceutical industry workers highlighted the need for practical and understandable instructions of various hand hygiene procedures, including the clarification of the possible technical mistakes. This became a ground for us to create individual master classes on hand hygiene for each cluster of healthcare workers. 

## Notes

### Competing interests

The authors declare that they have no competing interests.

### Acknowledgement

The study was supported by WYLAN, SME Ltd., Kiev, Ukraine and Desant Ltd., Kiev, Ukraine. 

## Figures and Tables

**Table 1:**
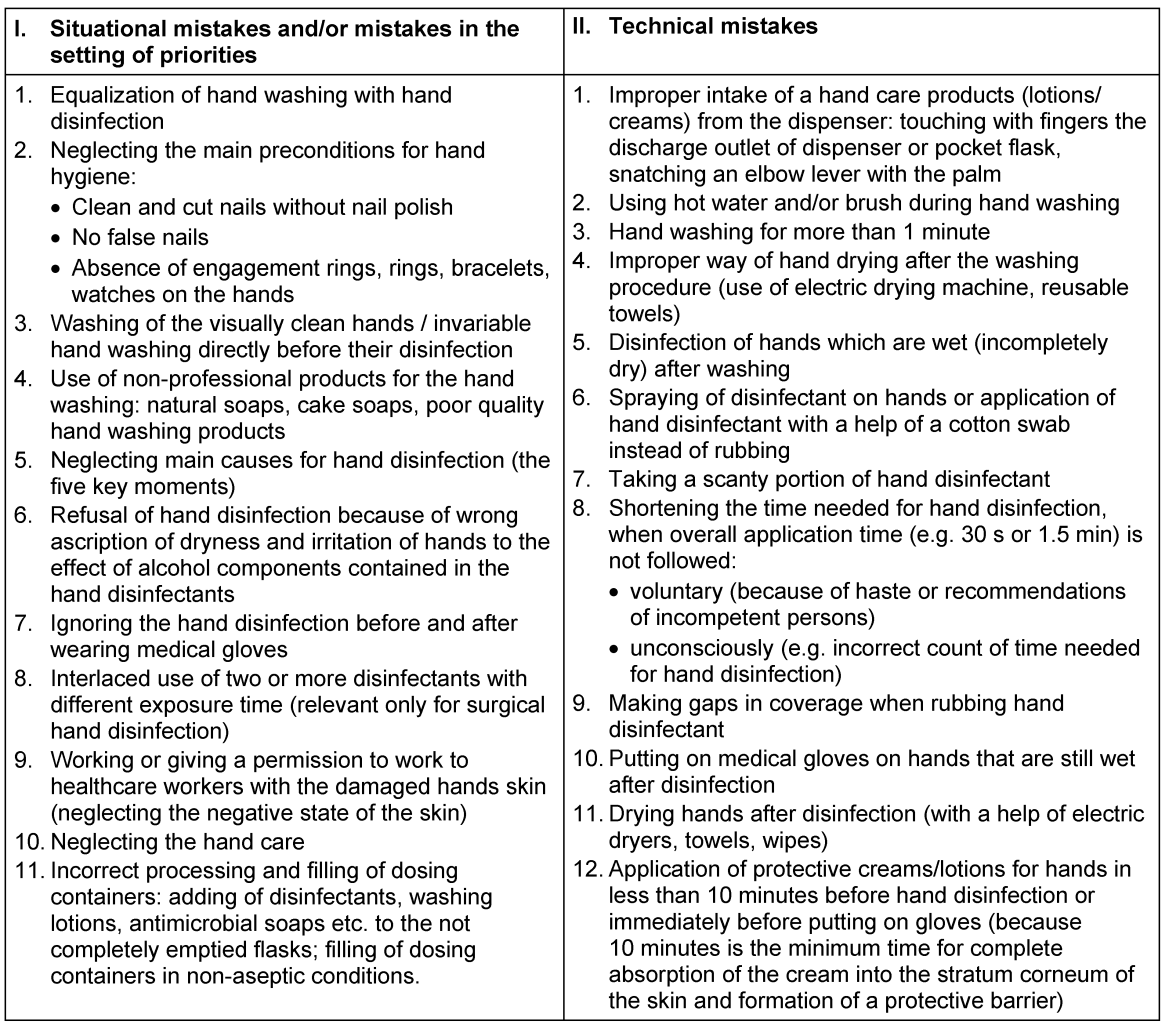
Systemic mistakes in hand hygiene practice

**Table 2:**
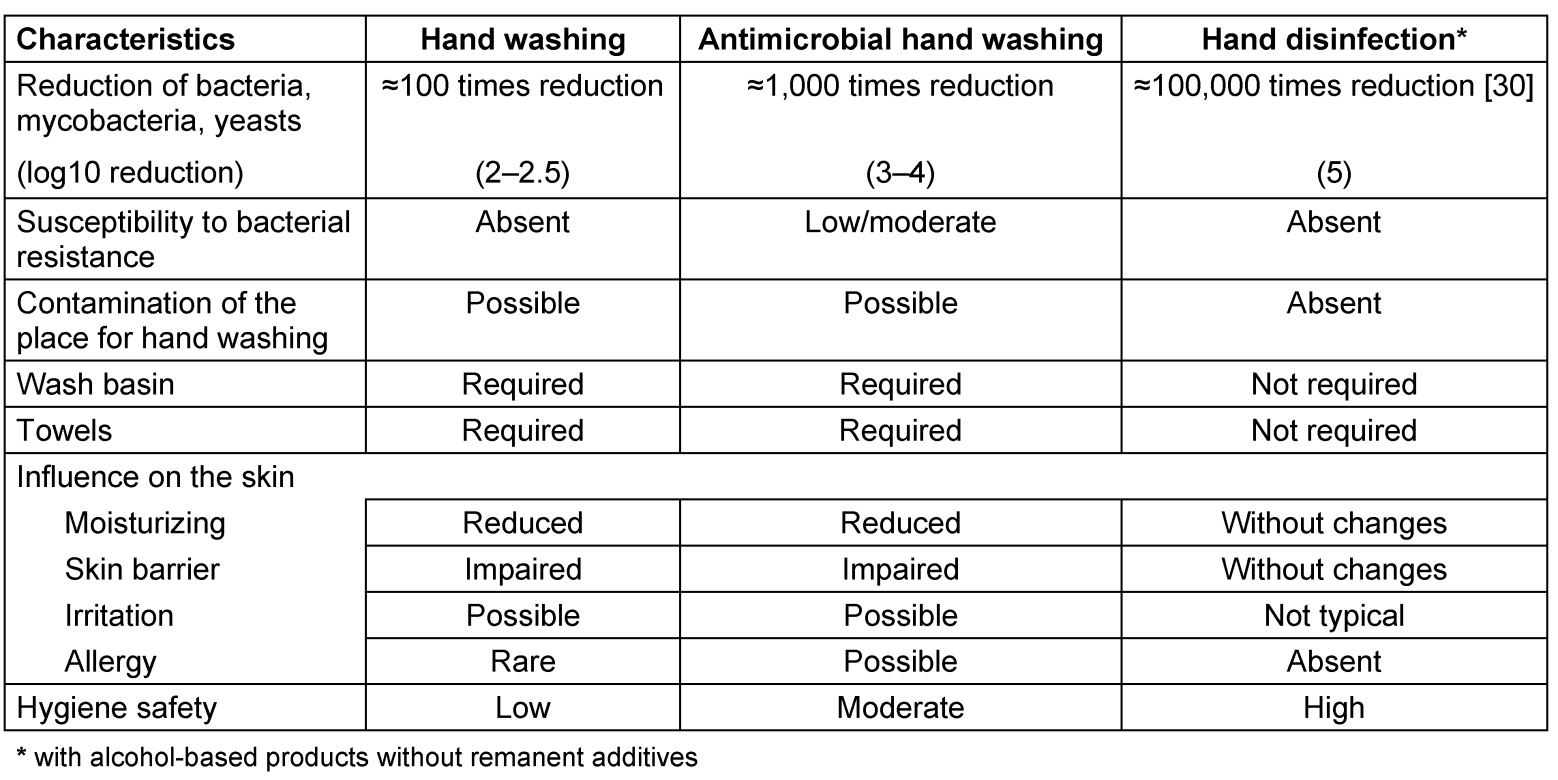
Comparative analysis of hand hygiene procedures

**Table 3:**
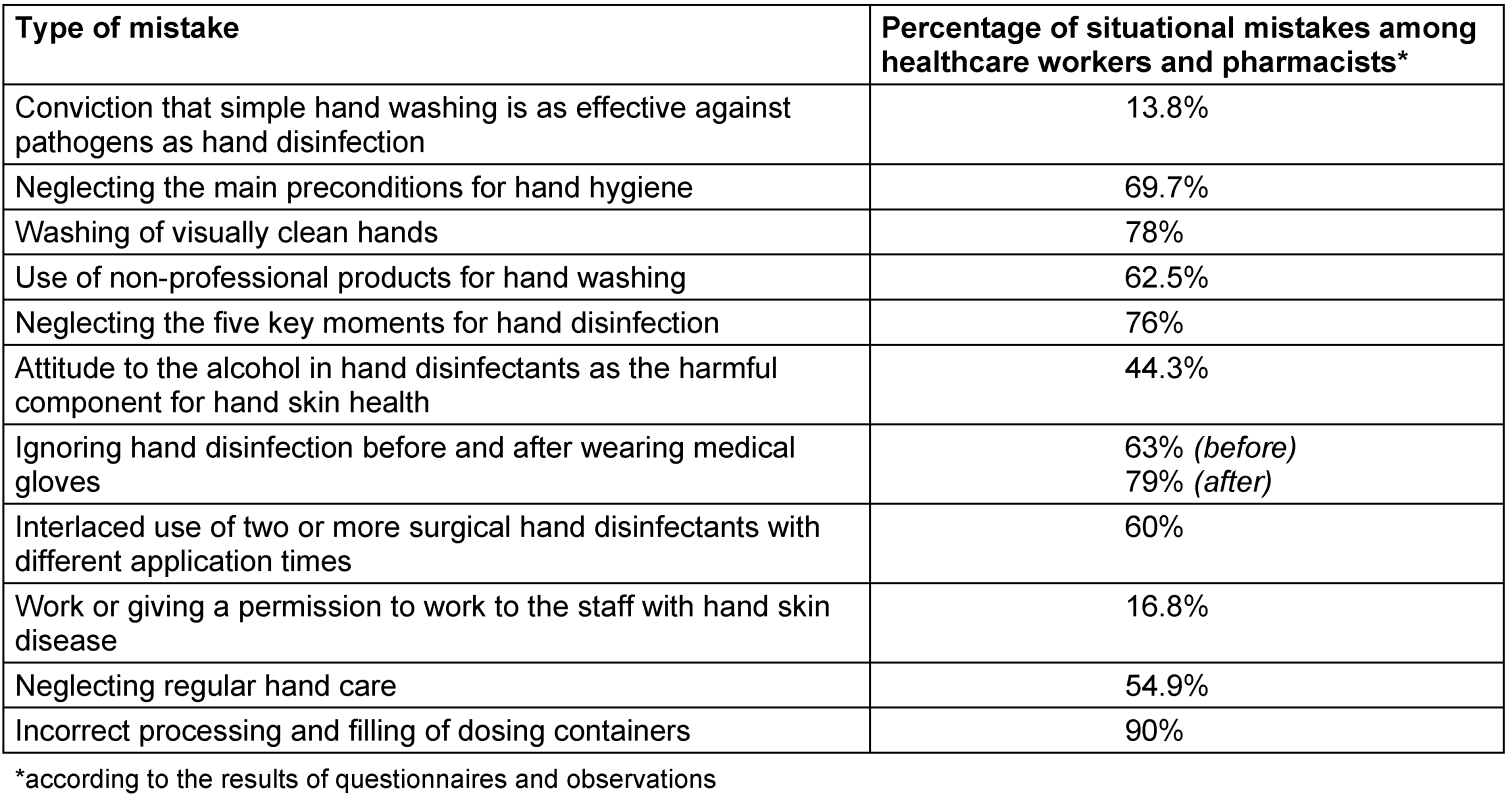
Percentage of situational mistakes among medical and pharmaceutical staff
